# Cancer Care After the 2017 Central Mexico Earthquake

**DOI:** 10.1200/JGO.18.00146

**Published:** 2018-08-15

**Authors:** Jacqueline Alcalde-Castro, Thierry Hernández-Gilsoul, Ismael Domínguez-Rosado, Yanin Chavarri-Guerra, Enrique Soto-Perez-de-Celis

**Affiliations:** **All authors:** Instituto Nacional de Ciencias Médicas y Nutrición Salvador Zubirán, Tlalpan, México

## TO THE EDITOR:

Natural disasters are major geophysical (earthquakes, volcano eruptions) or weather-related (hurricanes, flooding) events that may cause loss of life and damage to infrastructure. Disasters test the resilience of health care systems and may hamper their ability to provide care for patients with noncommunicable diseases (NCDs) such as cancer.^1^ On September 19, 2017, a magnitude 7.1 earthquake hit Central Mexico, causing an unofficial death toll of 369 people, of whom 228 died in Mexico City.^2^ In addition, 83 hospitals or clinics in South-Central Mexico were reported to have major structural damage.^2^

**Fig 1:**
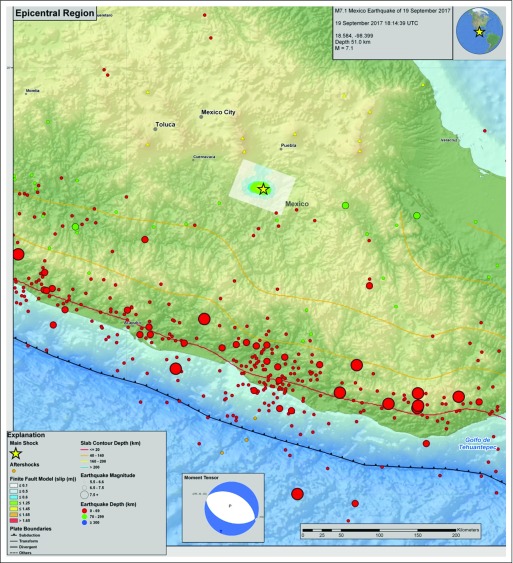
Location of the Epicenter of the 2017 Central Mexico Earthquake. Reproduced from the United States Geological Service (USGS) Web site, public domain.

Instituto Nacional de Ciencias Médicas y Nutrición Salvador Zubirán (INCMNSZ) is a 167-bed publicly funded academic third-level hospital treating approximately 600 new patients with cancer per year.^3^ INCMNSZ is located in southern Mexico City, approximately 110 km from the earthquake’s epicenter (Fig 1).^4^ After the earthquake, fissures were observed on the walls of the inpatient buildings (which house the operating rooms), prompting an evacuation. Stable patients, including those waiting for elective surgery, were discharged, and those needing to remain hospitalized were moved to the emergency department (ED) building, which suffered no damages. After comprehensive engineering analysis, the inpatient wards and operation rooms were found to have nonstructural damage, and the entire hospital was restored to full capacity within 6 weeks after the earthquake.

We studied the effect of the earthquake and the ensuing emergency situation on the treatment of patients with cancer at INCMNSZ. We reviewed charts of patients who were hospitalized on September 19, 2017, and identified those with a cancer diagnosis. We reviewed elective oncological surgical procedures that were rescheduled because of the earthquake, as well as visits to the ED by patients with cancer during the first month after the earthquake. The proportion of patients referred to other institutions, treatment delays due to referrals, and delays between planned and actual surgical dates were recorded. Outpatient visits, radiotherapy, and chemotherapy administration were not reviewed, because those facilities were not damaged.^5^ Descriptive statistics, including means, medians, ranges, and standard deviations, were used to analyze the data.

Of the 164 patients who were hospitalized on September 19, 2017, 36 had a diagnosis of cancer. Median age was 60 years (range, 24 to 78 years), and 23 (64%) were men. Eighteen (50%) had GI, 11 (30%) genitourinary, four (11%) gynecologic, and three (8%) other tumors. Most patients (47%; n = 17) had metastatic disease. The main reason for hospitalization was elective surgery (42%; n = 15), followed by infections (31%; n = 11). Eighteen patients (50%) were discharged from the hospital immediately after the earthquake, and three patients (8%) died during the first month after the earthquake.

Thirty oncological surgeries were rescheduled or canceled as a consequence of the earthquake. The characteristics of patients who had modifications in surgical planning are shown in Table 1. Twenty-four (80%) surgeries were rescheduled and six (20%) were cancelled. Nine of the surgical interventions had to be performed at other institutions. The median delay from planned to actual surgical date was 22.5 days (range, 3 to 130 days).

**Table 1:**
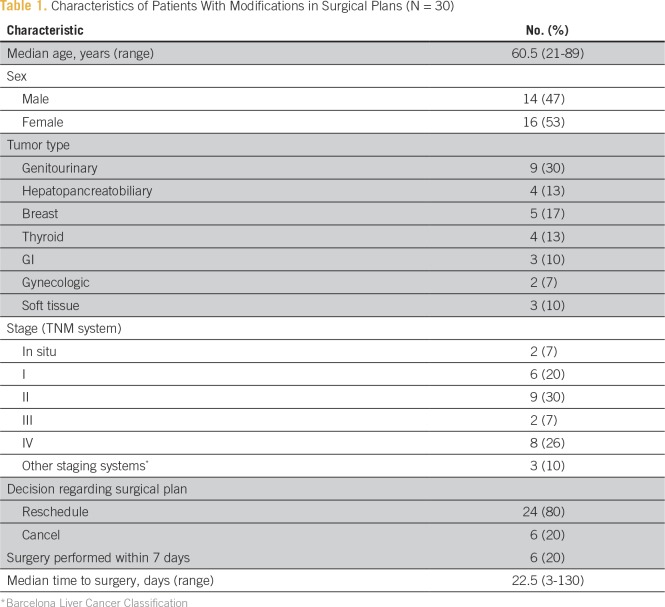
Characteristics of Patients With Modifications in Surgical Plans (N = 30)

The ED was visited by 2,196 patients in the first month after the earthquake, of whom 157 had a diagnosis of cancer (median age, 64 years; range, 23 to 91 years). The main reasons for ED visit among patients with cancer were infections (34%; n = 51), followed by renal/electrolyte disorders (11%; n = 17). No visits because of earthquake-related injuries were recorded. Ninety-eight patients (64%) were discharged from the ED, 40 (25%) were admitted at INCMNSZ, and 14 (9%) were referred to other institutions. A delay in oncological treatments directly related to the earthquake was found in nine (6%) patients.

After an earthquake evacuation, viability of the hospital dictum can be delayed, because engineering condemnation and functional damages may differ from initial inspections.^6^ Even though INCMNSZ was able to mount an effective surge capacity response and to repair damaged facilities promptly, a significant proportion of patients with cancer had treatment modifications. Although the small sample size and the heterogeneity of the population make it difficult to analyze survival outcomes, our results are an example of the effect natural disasters may have on the care of NCDs such as cancer. Health care systems of countries at high risk of natural disasters must ensure the continuity of care of patients with NCDs at times of emergency and create referral systems that can guarantee the completion of treatment in a prompt manner.^1^
